# Spontaneous hybridization and introgression between walleye (*Sander vitreus*) and sauger (*Sander canadensis*) in two large reservoirs: Insights from genotyping by sequencing

**DOI:** 10.1111/eva.13174

**Published:** 2020-12-14

**Authors:** Carly F. Graham, Rebecca L. Eberts, Una Goncin, Christopher M. Somers

**Affiliations:** ^1^ Department of Biology University of Regina Regina SK Canada; ^2^ Fish, Wildlife, and Lands Branch, Ministry of Environment Government of Saskatchewan Prince Albert SK Canada

**Keywords:** genotyping by sequencing, hybridization, introgression, sauger, walleye

## Abstract

Anthropogenic activities may facilitate undesirable hybridization and genomic introgression between fish species. Walleye (*Sander vitreus*) and sauger (*Sander canadensis*) are economically valuable freshwater species that can spontaneously hybridize in areas of sympatry. Levels of genomic introgression between walleye and sauger may be increased by modifications to waterbodies (e.g., reservoir development) and inadvertent propagation of hybrids in stocking programs. We used genotyping by sequencing (GBS) to examine 217 fish from two large reservoirs with mixed populations of walleye and sauger in Saskatchewan, Canada (Lake Diefenbaker, Tobin Lake). Analyses with 20,038 (r90) and 478 (r100) single nucleotide polymorphisms clearly resolved walleye and sauger, and classified hybrids with high confidence. F_1_, F_2_, and multigeneration hybrids were detected in Lake Diefenbaker, indicating potentially high levels of genomic introgression. In contrast, only F_1_ hybrids were detected in Tobin Lake. Field classification of fish was unreliable; 7% of fish were misidentified based on broad species categories. Important for activities such as brood stock selection, 12 of 173 (7%) fish field identified as pure walleye, and one of 24 (4%) identified as pure sauger were actually hybrids. In addition, two of 15 (13%) field‐identified hybrids were actually pure walleye or sauger. We conclude that hybridization and introgression are occurring in Saskatchewan reservoirs and that caution is warranted when using these populations in stocking programs. GBS offers a powerful and flexible tool for examining hybridization without preidentification of informative loci, eliminating some of the key challenges associated with other marker types.

## 1 INTRODUCTION

Hybridization and introgression, resulting in gene flow between species, can have conservation and management implications. Natural hybridization is an important evolutionary process that occurs in a variety of contexts (Caniglia et al., 2020; Hedrick, 2013; Marques et al., 2019; Mitchell et al., 2019). Conversely, anthropogenic hybridization is the result of human disturbances, such as intentional admixture, translocations, habitat modifications, and climate change, which can lead to hybridization that would not normally occur (reviewed by Caniglia et al., 2020; McFarlane & Pemberton, 2019). Anthropogenic hybridization can lead to introgression, which can be a concern because it can impact local and global biodiversity (Gilman & Behm, 2011). Further, human‐mediated hybridization can cause problems for native species, such as outbreeding depression, genetic swamping, and potentially extinction or extirpation (Gilman & Behm, 2011; McFarlane & Pemberton, 2019; Rhymer & Simberloff, 1996; Todesco et al., 2016). Anthropogenic hybridization has frequently been studied in fish as a result of concern generated by stocking programs (Dierking et al., 2014; Harbicht et al., 2014; Huuskonen et al., 2017), introduction of non‐native species (Boyer et al., 2008; Lamaze et al., 2012), and habitat alterations (Huuskonen et al., 2017). It is important to understand where and how humans artificially influence hybridization to help maintain biodiversity and fisheries productivity.

In North America, female walleye (*Sander vitreus*) and male sauger (*Sander canadensis*) can hybridize to produce viable “saugeye” (Billington et al., 1997; Lynch et al., 1982; Ward & Berry, 1995). Sauger are often found in more turbid, riverine environments compared with the lacustrine habitats preferred by walleye, but overlap in spawning times and locations occurs in some regions and can lead to the spontaneous generation of wild hybrids (Bozek, Baccante, et al., 2011; Bozek, Haxton, et al., 2011; Clayton et al., 1973; Nelson & Walburg, 1977; Stroud, 1948). Hybridization is more common in artificial or altered systems (e.g., reservoirs) and where parental species did not naturally co‐occur (reviewed by Billington & Sloss, 2011). First‐generation (F_1_) hybrids have been found in a wide variety of systems, but second‐generation hybrids (F_2_) are rare due to the low reproductive success of F_1_ × F_1_ crosses; however, F_1_ hybrids can successfully backcross with either parental species (Bingham et al., 2012; Fiss et al., 1997). In addition to naturally occurring hybrids, F_1_ saugeye have been artificially generated in hatcheries for targeted stocking programs for the purposes of creating put‐and‐take recreational fisheries, due to their faster growth rates in suboptimal habitats (Boxrucker, 2002; Galiant et al., 2002; Lynch et al., 1982; Malison et al., 1990; Pope et al., 1996; Siegwarth & Summerfelt, 1993; Stahl et al., 1996). However, little is known about the factors that influence hybridization frequency, or introgression between the species (multigenerational hybrids).

Spontaneous generation of saugeye creates potential fisheries management concerns in two major ways: (a) potentially reduced population productivity in areas with high percentages of F_1_ saugeye due to competition with parental species; and (b) reduced fitness of backcrossed individuals and genomic introgression between species (Billington et al., 1997; Epifanio & Waples, 2015; Fiss et al., 1997; Hearn, 1986; Koch et al., 2018; Quist et al., 2010; White et al., 2005). These issues have been documented in other species that hybridize, such as Paiute cutthroat trout (*Oncorhynchus clarkia seleniris*) × rainbow trout (*Oncorhynchus mykiss*; Busack & Gall, 1981), westslope cutthroat trout (*Oncorhynchus clarki lewisi*) × rainbow trout (Weigel et al., 2003), native and non‐native rainbow trout (Campton & Johnston, 1985), native and non‐native brown trout (*Salmo trutta*; Araguas et al., 2004and smallmouth bass (*Micropterus dolomieu*) × spotted bass (*Micropterus punctulatus*; Pierce & Van Den Avyle, 1997). To circumvent this issue, hatchery programs may use sterile triploid hybrids, including saugeye, that are unable to backcross with parental species, to facilitate more controlled stocking programs (Czensy et al., 2002; Fetherman et al., 2015; Garcia‐Abiado et al., 1999, 2002; Kerby et al., 2002; Koch et al., 2018; Quist et al., 2010; Willis et al., 1994). However, spontaneous hybridization in the wild is much more difficult to predict or control. Stocking programs that collect spawning materials from wild walleye or sauger are particularly at risk for inadvertently propagating saugeye through the artificial culture and dispersal of stock into other waterbodies due to the potential misidentification of hybrid individuals used as broodstock (Billington et al., 1997; Hartman et al., 2019; Van Zee et al., 1996; Ward & Berry, 1995; White et al., 2005).

In situations where walleye and sauger are sympatric or syntopic, identification of saugeye and estimation of hybridization rates are important for fisheries managers. A variety of markers have been used to identify saugeye and have estimated hybridization frequencies ranging from 0% to 39% in various waterbodies (reviewed by Billington & Sloss, 2011). The most readily available tool is field identification based on morphological features; however, the possibility for saugeye to present a mosaic of phenotypic features renders field markers unreliable (Billington et al., 1997; Flammang & Willis, 1993; Van Zee et al., 1996; Ward & Berry, 1995; White et al., 2005). Genetic identification tools used previously include protein electrophoresis (allozymes; Billington et al., 1997; Fiss et al., 1997; Flammang & Willis, 1993; Graeb et al., 2010; Krueger et al., 1997; Van Zee et al., 1996), mtDNA restriction fragment length polymorphisms (RFLP; White et al., 2005), fluorescent randomly amplified polymorphic DNA markers (FRAPD; Sovic et al., 2012), and microsatellites (Bingham et al., 2012; White et al., 2005, 2012). However, the limited number of genetic loci assessed with these methods may reduce the ability to correctly assign species categories and limit the potential to identify hybrids with low levels of admixture (Billington et al., 1997; Bingham et al., 2012; Boecklen & Howard, 1997; Twyford & Ennos, 2012; Vaha & Primmer, 2006). Further, the diagnostic nature of these markers may be population‐specific, and some can be difficult to replicate across different laboratories (e.g., microsatellite DNA; Fernandez et al., 2013; Stott et al., 2010; Vignal et al., 2002). Overall, the methods currently used to identify saugeye have some potentially important limitations and our understanding of spontaneous hybridization would be enhanced by a more powerful genomics approach.

High‐throughput DNA sequencing enables the screening of thousands of single nucleotide polymorphisms (SNPs) throughout the genome, resulting in much higher resolution for hybridization studies (Baird et al., 2008; Davey et al., 2011; Etter et al., 2011; Hohenlohe et al., 2011; Twyford & Ennos, 2012). There are several promising advantages to using SNPs for investigating hybridization and introgression, including their abundance throughout the genome in coding and noncoding regions, the ability for automated genotyping, and the ease of sharing large datasets among laboratories (Baird et al., 2008; Davey et al., 2011; Etter et al., 2011; Helyar et al., 2011; Schlotterer, 2004; Vignal et al., 2002). Reduced representation library (RRL) sequencing approaches, such as genotyping by sequencing (GBS), generate a subset of homologous loci that can provide broad coverage across the genome, and the ability to multiplex many individuals within a single sequencing run (Baird et al., 2008; Davey et al., 2011; Etter et al., 2011; Greminger et al., 2014; Van Tassell et al., 2008). This approach has been effectively used to detect hybridization and introgression in aquatic (Amish et al., 2012; Baird et al., 2008; Benestan et al., 2015; Hand et al., 2015; Hohenlohe et al., 2011) and terrestrial animals (Barley et al., 2019; Melville et al., 2017; Ottensburgh et al., 2017), as well as plant species (Eaton & Ree, 2013; Gramlich et al., 2018; Owens et al., 2016). To date, GBS has not been applied to studies of walleye–sauger hybridization.

Here, we use GBS and SNPs to examine spontaneous hybridization between walleye and sauger in two large reservoirs (Lake Diefenbaker and Tobin Lake) in the Saskatchewan River system of Saskatchewan, Canada. Little is known about the geographical distribution and frequency of hybridization in Saskatchewan; however, there is extensive range overlap between walleye and sauger, and saugeye have been identified morphologically in both reservoirs. These species are of principal interest given the high economic and cultural value of recreational walleye fisheries in the region (Fisheries & Oceans Canada, 2018; Saskatchewan Ministry of Environment, 2010). In addition, until 2013, the main source of walleye broodstock for the provincial stocking program came from Lake Diefenbaker, raising concern about the genetic integrity of stocked walleye. In 2004, hybridization frequencies in Lake Diefenbaker were estimated to be as high as 22% based on allozymes; however, broodstock collection was still considered feasible in certain areas of the lake (Billington et al., 2005). Given limited knowledge regarding spontaneous hybridization, our overarching goal was to harness the power of GBS and SNPs to identify saugeye. Our specific objectives were to: (a) use GBS and SNPs to classify fish from mixed populations of walleye and sauger without prior identification of informative loci; (b) assess our ability to correctly classify species based on morphology; and (c) apply the GBS approach to understand the extent of hybridization and introgression in two reservoirs in Saskatchewan, Canada. Our work provides insight into hybridization as a biological phenomenon, and the use of GBS as a powerful and flexible approach for identifying different levels of admixture among hybridizing species.

## 2 MATERIALS AND METHODS

### 2.1 Study sites

**FIGURE 1:**
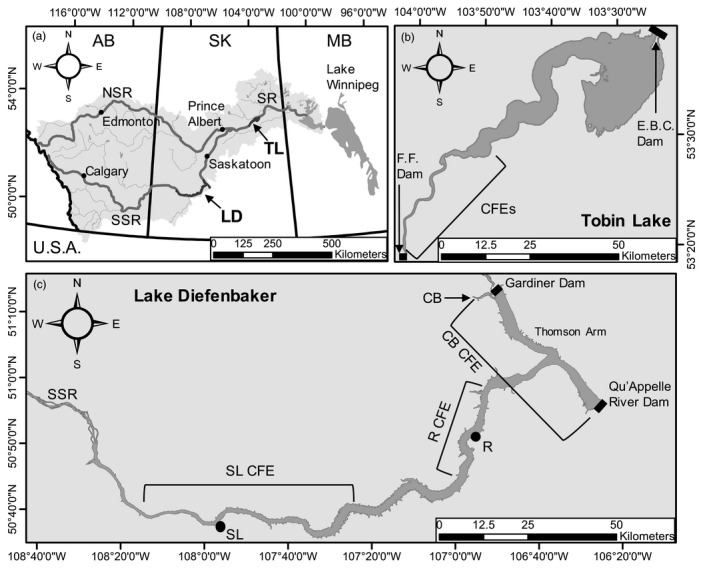
(a) Location of Lake Diefenbaker (LD) and Tobin Lake (TL) within Saskatchewan. Canada. The Saskatchewan River basin (light gray), South Saskatchewan River (SSR), North Saskatchewan River (NSR), Saskatchewan River (SR), and major cities are shown for reference. On Tobin Lake (b), fish were sampled from two CFEs near Nipawin, which have the same fishing boundaries in the river section (see bracket). Locations for the Francois Finlay Dam (F.F.) and E.B. Campbell Dam (E.B.C.) are shown. On Lake Diefenbaker (c), fish were sampled from three CFEs centered around Saskatchewan Landing (SL), Riverhurst (R), and Coteau Bay (CB) with distinct fishing boundaries (see brackets). Locations of the Gardiner Dam and Qu'Appelle Dam are shown

Lake Diefenbaker (51°01′53″N 106°50′09″W) is a large (surface area 394 km^2^), deep (mean depth = 22 m; max. depth = 60 m; North et al., 2015) reservoir of the South Saskatchewan River spanning 230 km across the Saskatchewan prairie (Water Security Agency, 2012; Figure 1). The reservoir was created in 1967 by the construction of the Gardiner and Qu'Appelle River Dams in the South Saskatchewan River and Qu'Appelle River, respectively (Water Security Agency, 2012; Figure 1). The reservoir is characterized by a transition from shallow, riverine habitat at the upstream end to a deep lacustrine habitat in the Thomson Arm of the downstream end, with a sandy shoreline consisting of numerous tributaries (North et al., 2015; Sadeghian et al., 2015). The reservoir is a critical water supply for Saskatchewan and provides water allocation for irrigation, power, and municipal and industrial uses (Saskatchewan Water Security Agency, 2012). Lake Diefenbaker supports at least 26 native and stocked fish species, including self‐sustaining populations of both walleye and sauger (Saskatchewan Water Security Agency, 2012). In addition to its purpose for water allocation, Lake Diefenbaker is managed as a recreational fishery and is one of the most popular destinations for domestic and international tourism in the province.

The Saskatchewan Ministry of Environment has been actively stocking millions of walleye fry annually into lakes across a variety of Saskatchewan water systems since 1950 (Wallace, 2004). For example, in 2019 a total of 53 waterbodies were stocked with over 10.1 million walleye fry (Saskatchewan Ministry of Environment, 2019). This stocking effort generally targets lakes that do not naturally sustain walleye, but in some circumstances, supplemental stocking has been used to augment natural productivity. Until 2012, Coteau Bay on Lake Diefenbaker (Figure 1c) was the main source for walleye eggs, with up to 100 million eggs collected (Wallace, 2004). In 2004, concern for potential hybridization between walleye and sauger in Lake Diefenbaker and the spread of hybrids through stocking prompted the Saskatchewan Ministry of Environment to commission a study on hybridization based on allozymes. Overall hybridization frequency based on fish collected in a gill net survey was estimated to be 21% based on four diagnostic allozyme loci, although with this few markers there is a 6.25% chance that introgressed alleles were missed (Billington et al., 2005). The highest rate of hybridization was detected in the western upstream riverine area of Lake Diefenbaker (i.e., Saskatchewan Landing). Based on uncertainty about the genetic status of fish in Lake Diefenbaker and specifically Coteau Bay, broodstock collections for the stocking program were switched to alternate locations containing only walleye (no sympatric sauger). However, the Coteau Bay location remains of interest for future stocking efforts, and there is a need to understand hybridization between walleye and sauger more comprehensively in Lake Diefenbaker.

Tobin Lake (53°35′N, 103°30′W) is a large reservoir (surface area 228 km^2^) formed by the impoundment of the Saskatchewan River by the E.B. Campbell Dam (built in 1963), and farther upstream by the Francois Finlay dam (built in 1985; Figure 1; Warwick & Tisdale, 1988). Tobin Lake is also characterized by a transition of shallow remnant riverine habitat upstream to deeper lacustrine habitat downstream; however, the reservoir is much shallower (mean depth = 8 m, max depth = 26.4 m) overall than Lake Diefenbaker and is primarily used for hydroelectric power production. Tobin Lake supports at least 25 different species of fish, including walleye and sauger, and is considered one of the most valuable trophy fisheries in Saskatchewan (Orr, 1993). The recreational fishery on Tobin Lake is a major draw for anglers from both Canada and the USA. In a population assessment in 2018, the Saskatchewan Ministry of Environment fisheries staff morphologically identified six suspected saugeye, approximately 1% of the *Sander* spp. collected (R. Eberts, Saskatchewan Ministry of Environment, unpublished data); however, no genetic survey has been completed for that lake. The frequency of hybrids and backcrosses in the Tobin Lake system is of interest as a comparison to Lake Diefenbaker, and for a basic understanding of conditions that facilitate spontaneous hybridization. Both Lake Diefenbaker and Tobin Lake are large reservoirs where riverine and lake systems merge as a result of anthropogenic water management, which may be ideal conditions for hybridization of walleye and sauger (Bellgraph et al., 2008; Gangl et al., 2000).

### 2.2 Sample collection, DNA isolation, and sequencing

Walleye, sauger, and saugeye tissue samples were collected from both Lake Diefenbaker and Tobin Lake during competitive fishing events (CFE) during 2014–2016 open water seasons. Pectoral fin tissue (approximately 5 × 10 mm) was collected for DNA extraction from live‐released fish as they were brought to central weigh stations on shore. Fin clips were stored in lysis buffer (4.0 M urea/0.2 M NaCl/0.1 M Tris‐HCl, pH 8.0/0.5% *n*‐lauroylsarcosine/0.1 M 1,2‐cyclo‐hexanediamine) at 4°C until DNA extraction. On Lake Diefenbaker, fish were sampled from CFEs located at Coteau Bay (May 2014), Riverhurst (June 2014; 2016), and Saskatchewan Landing (July 2014; 2016), covering a large portion of the reservoir (Figure 1). On Tobin Lake, fish were sampled from CFEs out of Nipawin, which cover only the riverine portion of the reservoir (August, October 2014 and October 2016). On each day of the 2‐day CFEs, angling teams weighed in a total of five fish each at the conclusion of fishing (up to 160 teams per event). These fish were presented at the weigh station in plastic tubs; we intercepted a random subset of tubs at each CFE and collected fin clips from live fish. In each case, we inspected the tub of fish and sampled two walleye; if sauger or intermediate fish were present, we also sampled from that individual or individuals (major field markings used described by Billington et al. (1997)). Correspondingly, at each CFE we collected fin clips from hundreds of walleye, but only a small number of sauger or phenotypic intermediates, which were weighed‐in much less frequently. It is important to note that because fish were collected from CFEs, the data are based solely on individuals susceptible to angling. Consequently, hybridization and introgression levels may not be completely representative of the general populations in each reservoir.

During our field sampling, we accumulated a large tissue archive from CFEs on both study lakes. From these, a subset of 217 fish was prepared for GBS (Table 1). Samples were chosen for sequencing based on DNA quality (largest fragments present) and sample site to ensure that multiple individuals were sequenced from each location. DNA was extracted from fin clips using a Genomic DNA Isolation Kit following the manufacturer's guidelines (Norgen Biotek Corp.). Proteinase K digestion was extended to 12 hr at 55°C, and fin clips were treated with RNAse A (Qiagen Inc.). DNA concentration was then quantified using a Qubit 2.0 fluorometer (Life Technologies Inc.), and the quality was assessed using a 1% agarose gel (E‐Gel; Invitrogen). Although fin clips were collected from live fish and immediately preserved, there was still evidence of mild to moderate levels of shearing in the extracted DNA. To target an optimal number of SNPs per individual (see Graham et al., 2015), samples with DNA fragments ranging between 3 and 10 kb were retained for sequencing and those with higher levels of degradation were discarded.

**TABLE 1:** Collection data for 217 walleye (*Sander vitreus*), sauger (*Sander canadensis*) and putative hybrid saugeye from Lake Diefenbaker and Tobin Lake

Lake	Sample site	Code	Walleye	Sauger	Saugeye
Lake Diefenbaker		LD	86	12	15
	Coteau Bay	CB	15	0	0
	Riverhurst	R	42	5	3
	Saskatchewan Landing	SL	29	7	12
Tobin Lake	Nipawin	TL	92	12	0
Total			178	24	15

Note

Fish were characterized in the field based on phenotypic markings.

Samples were prepared for sequencing by the Genomics Analysis Core Facility at the Institute for Integrative and Systems Biology (IBIS, University de Laval) according to a modified GBS procedure developed by Mascher et al. (2013). Briefly, libraries were prepared using *PstI* and *MspI* restriction enzymes, and unique P1 adaptors, each containing in‐line barcodes for individual identification, were ligated to the DNA fragments. Individuals were then pooled, and fragments between 150 and 200 base pairs were isolated. Adapter‐ligated, size‐selected fragments were sequenced on an Ion Proton using 11 chips with the Ion PI™ Chip Kit v3 (Thermo Fisher Scientific), which has the capacity for 60–80 million reads (Recknagel et al., 2015).

### 2.3 Data analysis

#### 2.3.1 Genotyping and basic statistics

Raw reads were first visualized in FASTQC (Andrews, 2010) and then processed using STACKS version 2.3e (Catchen et al., 2011, 2013). The *process_radtags* script was used to remove any reads with uncalled bases, discard reads with an average quality score below Q10, and truncate the reads to 100 base pairs. Following quality filtering, the optimal distance allowed between stacks (‐M in *ustacks*), the minimum sequencing depth (‐m in *ustacks*), and the number of mismatches allowed between sample loci (‐n in *cstacks*) were optimized using the r80 rule as recommended by Paris et al. (2017). Optimized parameters for this study were determined to be *M* = 1, *m* = 3, and *n* = 2. The *ustacks* script was run using a minimum sequencing depth of 3 (‐m), a maximum distance of 1 bp allowed between stacks (‐M), and a maximum distance of 3 bp allowed to align secondary reads. The deleveraging algorithm was also enabled to resolve overmerged tags, while gapped alignments were disabled. Following *ustacks*, a catalog of loci was generated using 27 walleye and 17 sauger from across all of the sample sites with two mismatches allowed between sample loci, as determined above. Samples with an average number of reads were selected to be included in the catalog in order to reduce complexity, while still capturing the genetic diversity of each species. Following generation of the catalog, individual stacks were searched against the catalog using the *sstacks* script. The *tsv2bam* script was then run to transpose the data, followed by the *gstacks* script. Finally, the *populations* script was run to export SNPs. In order to avoid bias in missing data (see Graham, Boreham, et al., 2020), a populations map with no population designations was used (‐M), with the first SNP of each locus. Two datasets were generated where the loci had to be present in: (a) 90% of the individuals and (b) 100% of the individuals (‐r). The r90 dataset was used to investigate population structure both within and between species, while the r100 dataset was used to examine differentiation between species while generating a smaller group of candidate loci for assessment of genetic status (hybrid vs. pure).

The influence of missing data on the r90 dataset was examined using the *missing_visualization()* function within the *grur* R package (Gosselin & Archer, 2019; R Core Team, 2019). An isolation‐by‐missingness (IBM) plot was generated based on the presence/absence of genotypes within individuals across sample sites. Following the analysis of missing data, loci were checked for conformation to Hardy–Weinberg equilibrium (HWE; *p* < 0.05) using the *filter_hwe()* function in the *radiator* package (Gosselin & Archer, 2019). Loci that did not conform to HWE in two of the three species categories were used to create an exclusion list for analyses requiring HWE as an assumption.

Following quality control, basic population and species‐level statistics were calculated using both datasets. These basic statistics were calculated using groupings based on morphological identification for species. The nucleotide diversity (*π*) and the number of private alleles were calculated using the *pi()* and *private_alleles()* functions in *radiator*, respectively. The observed heterozygosity within subpopulations (*H*_O_), the expected heterozygosity within populations assuming HWE (*H*_S_), and the inbreeding coefficient (*G*_IS_) were calculated using GENODIVE (Meirmans & Van Tienderen, 2004). The expected heterozygosity (*H*_S_) within subpopulations is also known as the gene diversity and includes corrections for sampling bias (Nei, 1987). Individual heterozygosities were calculated using the *detect_mixed_genomes()* function in the *radiator* package (Gosselin & Archer, 2019). We then used a Kruskal–Wallis nonparametric analysis of variance (KW‐ANOVA) to statistically compare *H*_O_ values across species.

#### 2.3.2 Population subdivision analyses

Three different population structure analyses were then performed on each dataset: (a) fixation indices (*F*_ST_); (b) a maximum‐likelihood approach (ADMIXTURE); and (c) ordination (DAPC). The approaches using fixation indices and maximum likelihoods have underlying assumptions of HWE, so loci identified in the exclusion list mentioned above were removed from these analyses, while all loci were used in the ordination analysis. The *F*_ST_ analysis was conducted in GENODIVE by first computing a distance‐based matrix for all sites using AMOVA and then computing pairwise *F*_ST_ between sampling sites using 5000 permutations (Excoffier et al., 1992; Meirmans & van Tienderen, 2004). *F*_ST_ analysis requires a priori user‐defined groups for comparison; we used groups based on phenotype for this purpose. Significance values were adjusted for multiple comparisons using a false discovery rate (FDR) correction. The maximum‐likelihood approach was performed using ADMIXTURE (Alexander et al., 2009; Zhou et al., 2011). This program estimates the ancestry coefficient of each individual using a maximum‐likelihood approach followed by cross‐validation to determine the distinct populations (*K*; Alexander et al., 2009; Zhou et al., 2011). The R package *pophelper* was used to visualize ADMIXTURE results (Francis, 2017). The ordination analysis was conducted using discriminant analysis of principal components (DAPC) in *adegenet* (Jompart, 2008; Jompart & Ahmed, 2011). The DAPC plot was generated using the *optim_a_score()* function to determine the optimal number of principal components in order to avoid overfitting the data. ADMIXTURE and DAPC do not require a priori groupings for analysis.

#### 2.3.3 Identification of hybrids and genomic introgression

Our general approach involved the use of two unsupervised clustering programs, ADMIXTURE and NEWHYBRIDS, to group fish based solely on genotypes. No prior information about species (e.g., phenotype) was used in either analysis. We then compared genetic classifications to those based on identification using field markers. In ADMIXTURE, we used a value of *K* = 2 to identify pure individuals in two groups, and those that were intermediate to varying degrees. The power and accuracy of ADMIXTURE to identify pure and mixed individuals from differentiate hybrid classes was assessed by generating simulations of parental, F_1_, F_2_, and backcrosses using HYBRIDLAB v1.1 (Nielsen et al., 2006). Simulated allele frequencies were generated using walleye and sauger from the r100 dataset by crossing pure individuals identified in the NEWHYBRIDS analysis (see below). This simulation approach does not rely on potentially problematic misclassifications based on phenotype and removes circularity in the ADMIXTURE analysis by drawing on identification data from NEWHYBRIDS. The simulation program was used to generate 100 individuals of each pure and hybrid category. Following generation of the simulated populations, ADMIXTURE was run according to the parameters above to determine *Q*‐value thresholds for each hybrid category based on the fraction of the genome inherited from each ancestral genome (Alexander & Lange, 2011; Alexander et al., 2009; Anderson & Thompson, 2002; Vaha & Primmer, 2006).

Finally, a Bayesian statistical method was used to identify hybrids. The NEWHYBRIDS program computes the posterior probability that an individual in the sample belongs to each of the different hybrid classes based on genotype frequencies (Anderson & Thompson, 2002; do Prado et al., 2017; Vaha & Primmer, 2006). There were six different hybrid classes assessed in this analysis: (a) pure 1 represents pure sauger, (b) pure 2 represents pure walleye, (c) F_1_ are first filial hybrids created by pure 1 × pure 2, (d) F_2_ are second filial hybrids created from F_1_ × F_1_, and (e) BC1 and BC2 represent backcrossed individuals where F_1_ mate with pure 1 or pure 2, respectively. We used the command line version of NEWHYBRIDS with a burn‐in of 10,000 followed by 50,000 sweeps. This analysis was only run on the r100 dataset as it was unable to converge with the large number of loci in the r90 dataset.

## 3 RESULTS

### 3.1 Data analysis

#### 3.1.1 Genotyping and basic statistics

The sequencing resulted in a total of 676,280,651 reads with an average of 3,116,501 (*SD* ≅ 1,107,082) per individual. Following the *process_radtags* script, an average of 39,385 (*SD* ≅ 73,556) reads were removed due to missing radtags and 307,639 (*SD* ≅ 123,439) reads were removed due to low quality. This resulted in an average of 2,769,476 (*SD* ≅ 983,244) reads per individual remaining. Four individuals were then removed from further analysis because they had fewer than 1,000,000 reads. The final catalog contained 1,643,293 loci, which was generated using the 44 individuals specified above (27 walleye, 17 sauger). Following the *gstacks* script, there were a total of 644,856 loci genotyped with an average sequencing depth of 17.6× (*SD* ≅ 6.0×). The two datasets generated with the *populations* script resulted in 20,038 and 478 polymorphic SNPs in the r90 and r100 pools, respectively. One individual was identified as having more than 10% missing data with the IBM plot and was removed from further analyses. A total of 460 and five loci were identified as being out of HWE in two of the three species categories, creating a final dataset of 19,578 SNPs and 473 SNPs in HWE for the r90 and r100 datasets, respectively.

**FIGURE 2:**
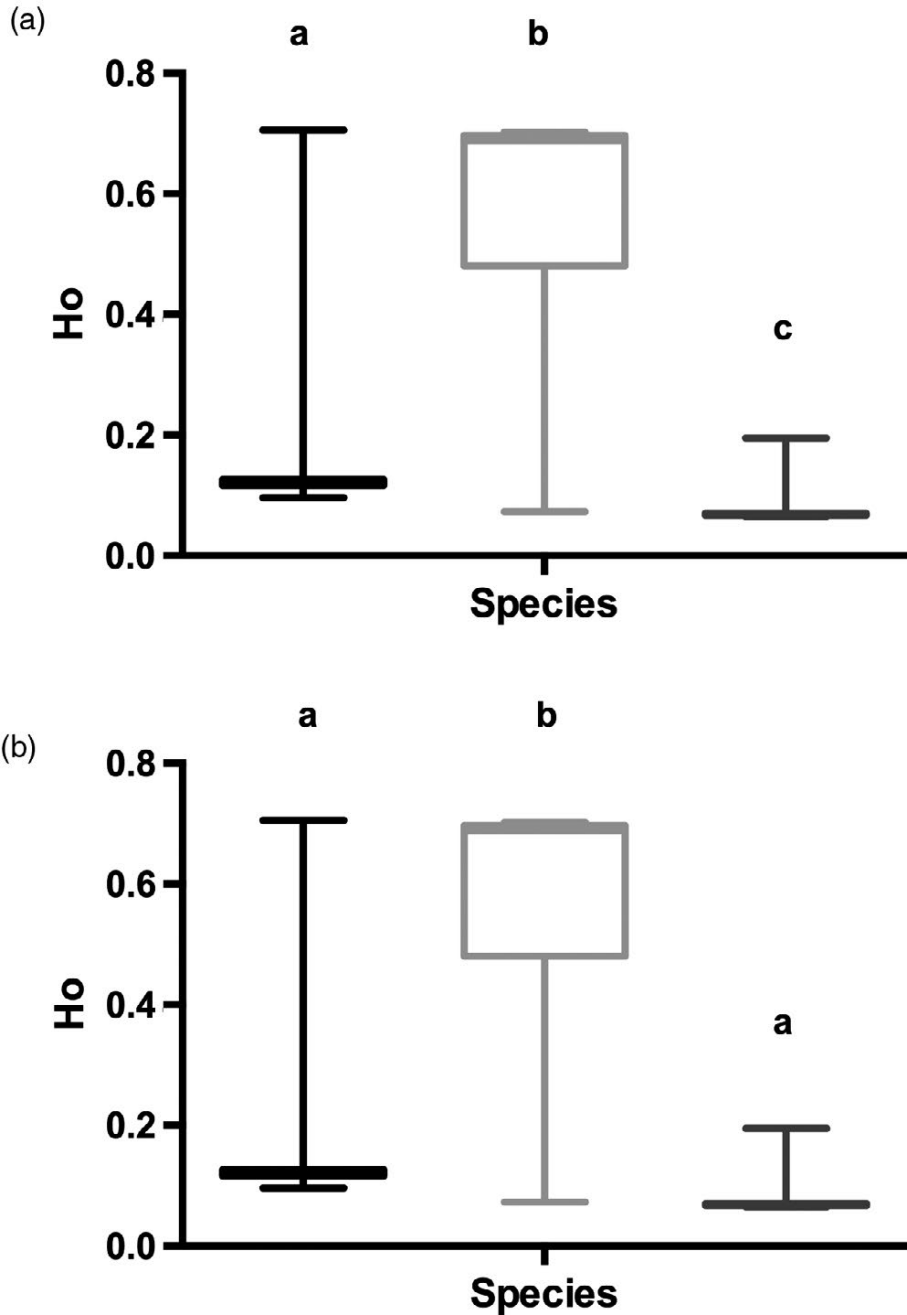
Individual observed heterozygosity (Ho) of each species. Heterozygosities were measured from each species in the dataset generated with 90% of the individuals (r90; a) and 100% of the individuals sampled (r100; b). The line in the middle of each box denotes the median, the box encompasses the first and third quartile of the data, while the horizontal lines above and below represent the maximum and minimum, respectively. The letters denote species or populations that are statistically different

Walleye had more private alleles in both datasets compared with sauger (Table 2). This is likely a result of sample size, where more private sauger alleles would likely be uncovered with more saugers sequenced. The average observed heterozygosity in saugeye was 4.0× and 7.6× greater in the r90 data and 5.2× and 6.2× greater in the r100 data than walleye and sauger, respectively (Table 2; Figure 2). Both walleye and sauger had very small *G*_IS_ values in both datasets, indicating neither an excess nor deficiency of heterozygotes (Table 2; Waples, 2015). The nucleotide diversity of saugeye was 2.80× and 5.45× larger in the r90 and 3.54× and 4.26× larger in the r100 dataset than that for walleye and sauger, respectively (Table 2).

**TABLE 2:** Basic species and population‐level statistics for the dataset generated with loci present in 90% of the individuals (r90) and 100% of the individuals (r100)

	*N*	PA	*H*_O_	*H*_S_	*G*_IS_	*π*
Walleye						
r90	173	485	0.143	0.144	0.008	0.000733
r100	173	14	0.116	0.113	−0.020	0.000577
Sauger						
r90	24	1	0.076	0.075	−0.005	0.000376
r100	24	0	0.098	0.096	−0.019	0.000479
Saugeye						
r90	15	0	0.578	—	—	0.00205
r100	15	0	0.603	—	—	0.00204

Note

Species groups were based on phenotype for these calculations. *N* is the number of individuals successfully genotyped that passed initial thresholds, PA is the number of private alleles present in each species, *H*_O_ is the observed heterozygosity, *H*_S_ is the expected heterozygosity under HWE, *G*_IS_ is the inbreeding coefficient, *π* is the nucleotide diversity.

#### 3.1.2 Population subdivision analyses

We used GENODIVE to determine pairwise *F*_ST_ values between each parental species category and sample site using both datasets (Tables S1 and S2). We detected strong significant differentiation across species with a very high *F*_ST_ value of 0.815 (*p* ≤ 0.001) between walleye and sauger in the r90 dataset and 0.848 (*p* ≤ 0.001) in the r100 dataset. This strong differentiation was detected with less sauger than walleye in the dataset, which may produce a less precise estimate of *F*_ST_ and other metrics. When comparing the differentiation across walleye sample sites, there was an average *F*_ST_ value of 0.030 (*SD* ≅ 0.020) in the r90 dataset and 0.045 (*SD* ≅ 0.038) in the r100 dataset (Tables S1 and S2). Sauger had average *F*_ST_ values of 0.012 (*SD* ≅ 0.008) across sites in the r90 dataset and 0.017 (*SD* ≅ 0.008) with average *p*‐values of 0.172 (*SD* ≅ 0.218) in the r100 datasets (Tables S1 and S2). Very little, although significant, differentiation was found within species across lakes with average *F*_ST_ values of 0.042 (*SD* ≅ 0.018) for walleye and 0.016 (*SD* ≅ 0.006) for sauger in the r90 dataset (Table S1). Similarly, *F*_ST_ values across lakes in the r100 dataset resulted in average values of 0.074 (*SD* ≅ 0.030) for walleye and 0.022 (*SD* ≅ 0.001) for sauger (Table S2).

**FIGURE 3:**
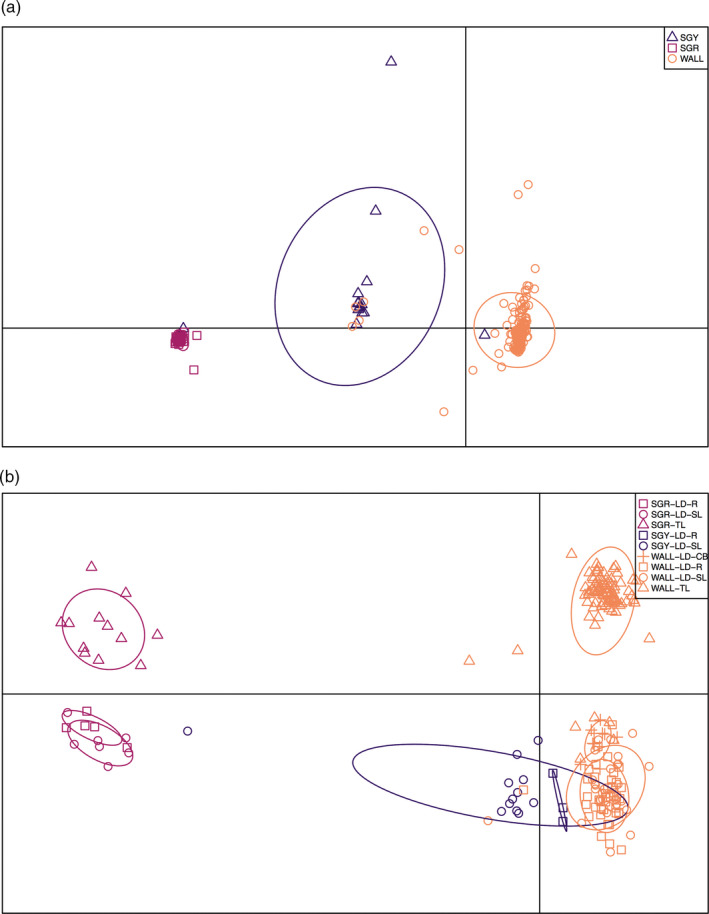
Discriminant analysis of principal components (DAPC) of the different species (a, b) and sample sites (c, d) in the r90 dataset (a, c) and r100 dataset (b, d). The DAPC analysis was run with 3 and 11 principal components in the species and sites analyses, respectively. Distinct ellipses indicate population differentiation. Site abbreviations can be found in Table 1

The DAPC analysis was run using six and nine principal components in the species‐level analyses with the r90 and r100 dataset, respectively, as determined using the *optim.a.score()* function (Figure 3a; Figure S1a). In the site‐specific analysis, 26 principal components were used with the r90 analysis and 13 were used with the r100 dataset (Figure 3b; Figure S1b). The inclusion of the optimal number of principal components resulted in 70.1% and 81.0% of the total variation retained in the r90 and r100 species‐level datasets, respectively (Figure 3a; Figure S1a). The sample site‐level analysis retained 75.0% in the r90 and 82.6% of the total variation in the r100 datasets (Figure 3b; Figure S1b). The first two discriminant functions represented 98.8% and 1.18% of the variation in the r90 dataset and 99.6% and 0.41% of the variation in the r100 datasets in the species‐level analysis (Figure 3a; Figure S1a). Similarly, the first two discriminant functions represented 61.0% and 19.4% of the variation in the r90 dataset and 77.7% and 18.6% of the variation in the r100 in the site‐level analyses (Figure 3b; Figure S1b). The assignment proportion was 0.967 in the r90 dataset and 0.972 in the r100 dataset at the species level. In the r90 dataset, five individuals morphologically identified as walleye were found to group with saugeye (W6442, W6511, W6723, W7012, and W7142), one saugeye grouped with walleye (H6383), and one saugeye grouped with sauger (H6631; Figure 3a). Using the r100 dataset, four individuals identified morphologically as walleye grouped with saugeye (W6442, W6723, W7012, and W7142), one saugeye grouped with walleye (H6383), and one saugeye grouped with sauger (H6631; Figure S1a). The assignment proportions for the sample site‐level analyses were 0.788 and 0.759 for the r90 and r100 datasets, respectively, at the sample site level (Figure 3; Figure S1).

**FIGURE 4:**
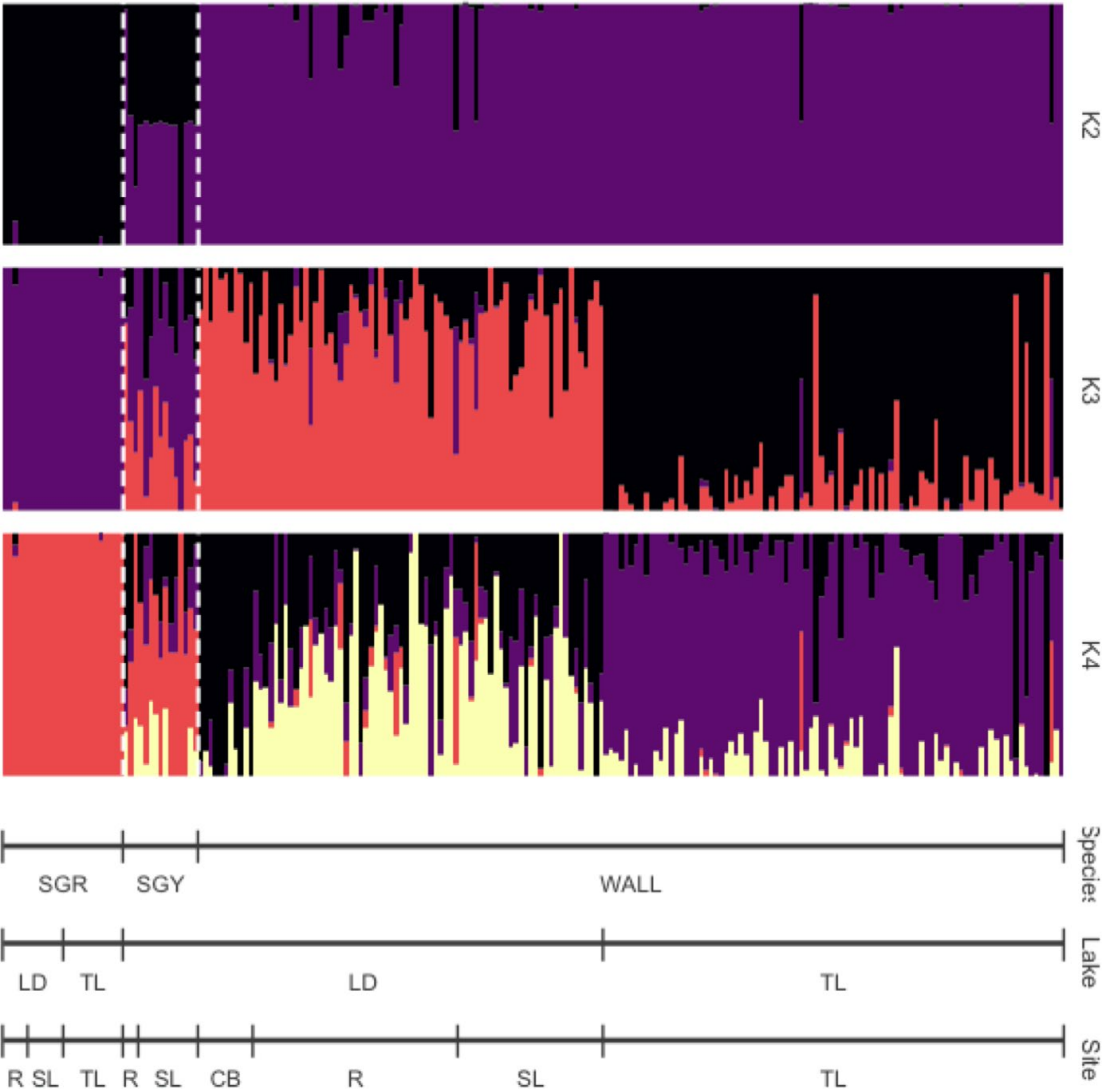
ADMIXTURE analysis across species from all sample sites in the (a) r90 and (b) r100 datasets. Each line represents an individual from the corresponding sample site. Site abbreviations can be found in Table 1. Asterisks above each figure represent misidentified individuals

ADMIXTURE was run on all sample sites and individuals using both r90 and r100 datasets (Figure 4; Figure S2). *K* = 3 had the lowest cross‐validation (CV) value in the r90 dataset with a value of 0.216, while *K* = 4 had a CV value of 0.217 (Figure 4). In the r100 dataset, *K* = 3 had the lowest CV value with 0.172 and *K* = 4 had a CV value of 0.176 (Figure S2). Population subdivision of walleye from different sites is clear with the *K* = 3 and *K* = 4 results, while we did not see structuring in sauger or saugeye with ADMIXTURE (Figure 4).

#### 3.1.3 Identification of hybrids and genomic introgression

ADMIXTURE output for *K* = 2 was used to resolve hybrids and parental species. The simulated data generated with HYBRIDLAB using pure individuals from NEWHYBRIDS indicated that with the r100 dataset, pure walleye had an average *Q*‐value of 0.99977 (*SD* ≅ 0.00109), and the average *Q*‐value for sauger was 0.00001 (*SD* ≅ 1.8729 × 10^–20^). F_1_ and F_2_ hybrids had *Q*‐values of 0.50063 (*SD* ≅ 0.00677) and 0.49998 (*SD* ≅ 0.01909), respectively. Due to the small difference in *Q*‐values between F_1_ and F_2_ hybrids, when estimating hybrid category, we combined these into F_1_/F_2_ hybrids, indicating that individuals could belong to either category. First‐generation backcrosses had *Q*‐values of 0.74960 (*SD* ≅ 0.01596) for walleye and 0.24928 (*SD* ≅ 0.01413) for sauger. Second‐generation backcrosses had *Q*‐values of 0.87562 (*SD* ≅ 0.01268) and 0.12496 (*SD* ≅ 0.01373), for walleye and sauger, respectively.

Based on the simulated data, hybrids were conservatively identified as having values 0.05 < *Q* < 0.95. ADMIXTURE results were identical for both r90 and r100 datasets. Overall, 15 of 212 (7%) fish were misidentified in the field based on morphology, resulting in a correct assignment value of 93% (Figure 4; Table 3; Data S1). One field‐identified sauger (S6462) caught at Riverhurst was genetically identified as a saugeye (Figure 4; Table 3). Two field‐identified saugeye were actually incorrect, with one from Riverhurst genetically identified as a walleye (H6383) and one from Saskatchewan Landing as a sauger (H6631; Figure 4; Table 3; Data S1). Twelve fish identified phenotypically as pure walleye in the field were genetically identified as saugeye: nine from Riverhurst, one from Saskatchewan Landing, and two from Tobin Lake (Figure 4; Table 3; Data S1). One of these walleye was genetically identified as an F_1_/F_2_ hybrid (W7012; Table 3; Data S1). Introgression was detected in eight of the phenotypically misidentified walleye from Riverhurst, with three of these individuals (W6412, W6442, and W6471) identified genetically as first‐generation backcrosses, and five (W6405, W6445, W6464, W6465, and W6472) as second‐generation backcrosses (Table 3; Data S1). One walleye from Saskatchewan Landing (W6511) and two from Tobin Lake (W6723 and W7142) were genetically identified as F_1_/F_2_ hybrids (Table 3; Data S1).

**TABLE 3:** Individual fish that were misidentified in the field based on morphological features, and their actual species category based on two genetic analyses, ADMIXTURE and NEWHYBRIDS

Individual	Field identification	Site	ADMIXTURE	Potential ADMIXTURE category	NEWHYBRIDS	*Q*‐value	Probability
H6383	SGY	LD‐R	WALL	Pure WALL	Pure WALL	0.96866	1.00000
H6384	SGY	LD‐R	SGY	F_1_/F_2_ SGY	F_2_ SGY	0.53253	1.00000
H6435	SGY	LD‐R	SGY	BC SGR	BC SGR	0.23324	1.00000
H6631	SGY	LD‐SL	SGR	Pure SGR	Pure SGR	0.00001	1.00000
S6462	SGR	LD‐R	SGY	BC2 SGR	BC SGR	0.09820	0.99897
W6405	WALL	LD‐R	SGY	BC2 WALL	Pure WALL	0.94624	1.00000
W6412	WALL	LD‐R	SGY	BC WALL	BC WALL	0.69408	1.00000
W6442	WALL	LD‐R	SGY	BC WALL	BC WALL	0.72292	1.00000
W6445	WALL	LD‐R	SGY	BC2 WALL	BC WALL	0.87289	1.00000
W6464	WALL	LD‐R	SGY	BC2 WALL	BC WALL	0.91959	0.99998
W6465	WALL	LD‐R	SGY	BC2 WALL	BC WALL	0.93833	1.00000
W6471	WALL	LD‐R	SGY	BC WALL	BC WALL	0.67549	1.00000
W6472	WALL	LD‐R	SGY	BC2 WALL	BC WALL	0.92637	1.00000
W6511	WALL	LD‐SL	SGY	F_1_/F_2_ SGY	F_1_ SGY	0.51160	1.00000
W6723	WALL	TL	SGY	F_1_/F_2_ SGY	F_1_ SGY	0.51360	1.00000
W7012	WALL	LD‐R	SGY	F_1_/F_2_ SGY	F_1_ SGY	0.48838	1.00000
W7142	WALL	TL	SGY	F_1_/F_2_ SGY	F_1_ SGY	0.50892	1.00000

Note

The ADMIXTURE column indicates what the individual was identified as using the ADMIXTURE program with the corresponding *Q*‐value in the specified column. The potential ADMIXTURE category is based on *Q*‐values with cutoffs from a simulation run in HYBRIDLAB, with F_1_/F_2_ indicating the individual could belong to either hybrid category. The NEWHYBRIDS column is based on Bayesian posterior probabilities with the full‐conditional probability of assignment in the probability column. F_1_ represents a cross between a pure walleye and a pure sauger, F_2_ represents the breeding of two F_1_ individuals, BC represents backcrossed individuals where an F_1_ breeds with a pure individual, and BC2 represents the breeding of a backcrossed individual with a pure parental individual. Site abbreviations can be found in Table 1. W6471 did not converge in Bayesian analyses and was therefore removed from the NEWHYBRIDS analysis.

**FIGURE 5:**
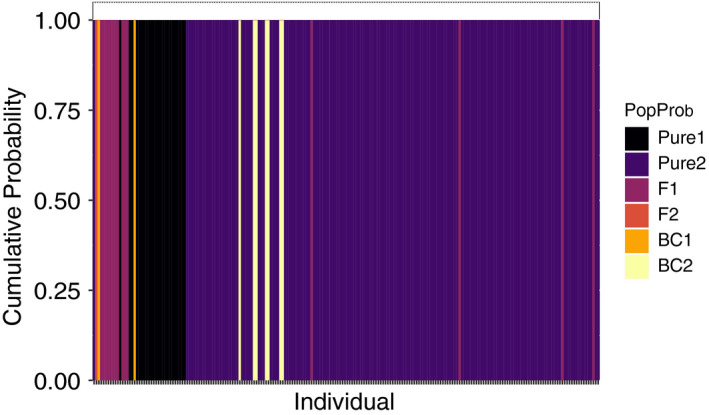
Results from the NEWHYBRIDS analysis using the r100 dataset. Each line represents an individual's posterior probability to a hybrid class. Pure 1 represents sauger, and pure 2 represents walleye. F_1_ individuals are filial 1 hybrids generated by pure 1 × pure 2. F_2_ individuals are filial 2 hybrids created by F_1_ × F_1_, and BC1 and BC2 represent backcrossed individuals where F_1_ mate with pure 1 or pure 2, respectively

Run independently, the NEWHYBRIDS analysis alone identified the same misidentified individuals as ADMIXTURE, except for one individual (W6405) identified as a pure walleye in NEWHYBRIDS and a potential second‐generation backcross in ADMIXTURE. A total of 14 of 212 (7%) fish were misidentified in the field, resulting in 93% correct assignment (Figure 5; Table 3; Data S1). One field‐identified sauger from Riverhurst (S6462) was genetically identified as a backcross (Figure 5; Table 3; Data S1). Two fish field identified as saugeye were misidentified, with one from Saskatchewan Landing genetically confirmed to be a sauger (H6631), and one from Riverhurst identified as a walleye (H6383), both of which were also identified in ADMIXTURE (Figure 5; Table 3; Data S1). Interestingly, one fish from Riverhurst field identified as a hybrid (H6384) was identified as an F_2_ hybrid in the NEWHYBRIDS analysis and one fish (H6435) was also identified to be a backcrossed sauger (Table 3; Data S1). A total of 11 fish identified as walleye in the field were actually hybrids. Four of the 11 fish were identified as F_1_: one from Riverhurst (W7012), one from Saskatchewan Landing (W6511), and two from Tobin Lake (W6723, W7142; Figure 5; Table 3; Data S1). Seven fish identified morphologically as walleye from Riverhurst were actually backcrossed walleye (W6412, W6442, W6445, W6464, W6465, W6471, and W6472; Figure 5; Table 3; Data S1).

Comparing the combined results of both analyses, ADMIXTURE and NEWHYBRIDS detected 17 of 214 (8%) individuals with incongruent identification in the field, with 16 of those present in both analyses (Table 3). It is important to note that any individual identified as belonging to a hybrid category based on either analysis method was considered a hybrid. There were two incongruencies across the different programs. One field‐identified walleye (W6405) was identified as a second‐generation backcross with ADMIXTURE but was identified as a pure walleye with NEWHYBRIDS. One field‐identified hybrid (H6384) was identified as an F_2_ hybrid with NEWHYBRIDS, but as an F_1_ based on *Q*‐values in ADMIXTURE. NEWHYBRIDS uses allele frequencies to determine specific hybrid classes, including designation of F_1_, F_2_, and backcrossed individuals. This approach resulted in better resolution when distinguishing between first‐ and second‐generation hybrids but was not able to resolve backcross generations (Table 3). In contrast, the simulations from HYBRIDLAB were unable to resolve F_1_ and F_2_ individuals but provided clear *Q*‐values associated with pure walleye and sauger, and first‐ and second‐generation backcrosses. Individuals identified as F_1_ in NEWHYBRIDS had average *Q*‐values of 0.50884 (*SD* ≅ 0.00778) in ADMIXTURE, while the F_2_ individual had a *Q*‐value of 0.53253. Overall, the analyses indicate that pure walleye were correctly identified 93% of the time based on morphological identification in the field. Tobin Lake had 97.8% correct assignment of pure walleye, compared with only 87.7% from Lake Diefenbaker. Combining the results from all analyses, the overall hidden introgression level in walleye based on our limited sampling was 4.6% across both reservoirs, and 9.9% and 0% in Lake Diefenbaker and Tobin Lake, respectively. Within Lake Diefenbaker, Riverhurst had a 19.5% level of hidden introgression, while zero hidden introgression was detected at Saskatchewan Landing and Coteau Bay.

## 4 DISCUSSION

The GBS approach we used worked well with unsupervised clustering methods to resolve different hybrid categories in two admixed populations of walleye and sauger without a priori knowledge of diagnostic loci. The power of GBS lies in the ability to genotype large numbers of loci across the genome, providing high resolution to detect hybridization and introgression (Ackiss et al., 2020; Allendorf et al., 2001; Boecklen & Howard, 1997; McFarlane & Pemberton, 2019; Melville et al., 2017; Miller, 2000; Randi, 2008; Young et al., 2001). This detection power offers a substantial improvement over more traditional markers, which likely underestimated introgression levels (Allendorf et al., 2001; Boecklen & Howard, 1997; Grabenstein & Taylor, 2018; Hohenlohe et al., 2013; Vaha & Primmer, 2006). Previous studies of hybridization have used similar RRL approaches for SNP genotyping, but took additional steps to identify a subset of informative loci to be genotyped using a more targeted approach (e.g., Hand et al., 2015; Hohenlohe et al., 2013; Pritchard et al., 2012; Pritchard et al., 2016). This strategy may limit the application of the identified SNPs to specific populations, similar to the limitations mentioned for other marker types (Hand et al., 2015; Pritchard et al., 2016; Wringe et al., 2019). Thus, rather than list a panel of specific loci for additional studies of walleye and sauger, we recommend that others apply the general approach of GBS for characterizing hybrids. GBS is very flexible across species and does not require initial investment in the development and quality control of diagnostic loci (Davey et al., 2011; Shafer et al., 2016). In addition, SNPs based on sequencing do not present the same challenges with scoring of alleles and are much more readily shared across laboratories and via archived data than previously used marker types (e.g., microsatellites, allozymes; Davey et al., 2011). The GBS approach should work broadly across different taxa and other species pairs, provided that the parental species are sufficiently divergent to offer clear resolution.

The ability to reliably resolve hybrids beyond first generation (F_1_) is critical for understanding introgression in areas with spontaneous hybridization. Unsupervised clustering approaches based on maximum likelihood (e.g., ADMIXTURE) and Bayesian probability (e.g., NEWHYBRIDS) are commonly used to identify and classify hybrids. The *Q*‐value thresholds for classifying individuals into different hybrid categories in ADMIXTURE have been examined using microsatellites, allozymes, and mtDNA in simulation studies (*reviewed in* McFarlane & Pemberton, 2019). *Q*‐value thresholds derived from simulations range from 0.8 to 0.999 based on the number of markers and divergence between parental species, with most studies with ~10 – 20 microsatellite loci using thresholds between 0.8 and 0.9 (Burgarella et al., 2009; do Prado et al., 2017; Marie et al., 2011; May‐McNally et al., 2015; Sacks et al., 2011; Sanz et al., 2009; Vaha & Primmer, 2006). The incorporation of more markers through a GBS approach reduces the error and maximizes detection of introgression (*reviewed by* McFarlane & Pemberton, 2019). However, *Q*‐value ranges have not been thoroughly investigated with GBS data without creating a panel of diagnostic loci (Randi, 2008; Vaha & Primmer, 2006). In our study, GBS enabled classification of parental species with very high confidence (*Q*‐values > 0.99), and our simulation in HYBRIDLAB suggested a range of 0.05 < *Q* < 0.95 to classify hybrids, similar to other GBS studies (Ackiss et al., 2020; Lavretsky et al., 2016). Importantly, this *Q*‐value range resulted in 94% agreement between ADMIXTURE and NEWHYBRIDS for identification of hybrids. However, there were some differences in several specific hybrid classifications, which has also been shown in previous SNP studies (Elliott & Russello, 2018; Gramlich et al., 2018; Pujolar et al., 2014). These differences result from the model used to determine hybrid classes, allowing NEWHYBRIDS to distinguish between F_1_, F_2,_ and backcrossed individuals (Anderson, 2009; Anderson & Thompson, 2002). Nevertheless, any individual identified as a hybrid using either program should be treated with caution when it comes to broodstock management in stocking programs.

Spontaneous hybridization of walleye and sauger is occurring in both of the major Saskatchewan River reservoirs we studied. In the case of Lake Diefenbaker, our finding confirms previous allozyme work (Billington et al., 2005), but genetically verified saugeye are a novel finding for Tobin Lake. Anthropogenic disturbances can break down reproductive isolation between species and may increase the rate of hybridization (*reviewed by* Rhymer & Simberloff, 1996; Todesco et al., 2016; McFarlane & Pemberton, 2019). Hydroelectric dams alter natural flow regimes, which cause significant alterations to native habitat, including the generation of artificial lakes (reservoirs) that replace reaches of the original river system (Carr et al., 2015). Over time, the reservoirs lose environmental heterogeneity and native habitat patterns, which can remove reproductive isolation between species (Hall et al., 2011; Hasselman et al., 2014; Seehausen et al., 2008). Dams have altered the habitat in the Saskatchewan River, resulting in the isolation of populations of walleye and sauger into smaller geographical sections. Further, reservoirs are characterized by a merging of riverine and lacustrine regions, creating areas of habitat transition, which likely influence interspecific interactions and facilitate hybridization (*reviewed in* Grabenstein & Taylor, 2018; Mandeville et al., 2019). The creation of reservoirs from the construction of dams in the Saskatchewan River has likely led to an increased level of hybridization due to the changes in habitat across the system.

The occurrence of hybridization and introgression varied substantially between reservoirs and was much more common in fish sampled from Lake Diefenbaker than Tobin Lake. Importantly, a single putative F_2_ hybrid and multiple generations of backcrosses with both walleye and sauger were detected in Lake Diefenbaker, while only F_1_ hybrids were found in Tobin Lake. It is important to note that although the F_2_ hybrid was only detected in one of the analyses, the high posterior probability in NEWHYBRIDS (1.000) likely means that it is a true second‐generation saugeye. This is only the second F_2_ saugeye reported in nature (see Fiss et al., 1997) and demonstrates viable F_1_ × F_1_ crosses in Lake Diefenbaker. Thus, F_1_ saugeye are reproducing in Lake Diefenbaker, causing introgression of the sauger genome into walleye, and vice versa. In contrast, we found no evidence of introgression in Tobin Lake. Multiple factors could contribute to the reservoir differences that we observed, including differences in relative abundance of parental species, overlap in habitat and spawning locations, and anthropogenic influences in each reservoir (Gilman & Behm, 2011; Grabenstein & Taylor, 2018; Scribner et al., 2001). Butt et al. (2017) found that ecological niche overlap between walleye and sauger differed substantially within the reservoirs, indicating there are underlying factors causing distinct interactions in these lakes. On a broader scale, these reservoirs are impoundments of distinct regions of the Saskatchewan River with different headwaters, habitat and productivity characteristics, and stressors, which may influence geographical patterns of hybridization. The South Saskatchewan River in particular (containing Lake Diefenbaker) is heavily altered due to its multiuse purpose (e.g., drinking water, power, and agricultural and industrial use), human population density, and associated land‐use activities in the Canadian prairies (Carr et al., 2015; Corkal et al., 2011; North et al., 2015). However, the factors that are most relevant for understanding hybridization (e.g., spawning event overlap; Hasselman et al., 2014; Mulfeld et al., 2009) are not well characterized in either reservoir or host river.

Hybridization and introgression in Lake Diefenbaker may vary based on location within the reservoir. Previous data based on allozymes from gill‐netted fish showed an east‐to‐west gradient of F_1_ hybrids, with the lowest level of hybridization detected in the northeast near Coteau Bay, and the highest near Saskatchewan Landing at the western end of the lake (Billington et al., 2005). The samples we collected from CFEs also showed within‐reservoir differences, with higher levels of hidden introgression (19.5%) in field‐identified walleye from the Riverhurst CFE, and zero hidden introgression at both the Saskatchewan Landing and Coteau Bay events. Although there was no hidden introgression detected at Saskatchewan Landing, multiple first‐generation hybrids were detected, whereas multiple generations of backcrosses and a second‐generation hybrid, F_2_, were detected at Riverhurst. There are major habitat differences among these areas of the lake, but it is uncertain whether apparent hybridization differences reflect the location of sampling, the time of year, and/or the characteristics of the particular fish susceptible to angling in the area at that time. Other studies have shown spatial gradients in hybridization frequency between lakes across large geographical regions, usually with high frequencies near the source reservoir (Albert et al., 2006; Graeb et al., 2010; Hargrove et al., 2019; Hasselman et al., 2014; Mandeville et al., 2017, 2019; Rudbridge & Taylor, 2005; Sotola et al., 2019). However, very few studies have detected hybridization differences based on sample site within a lake. Additional research is required to understand potential spatial structuring of introgression in Lake Diefenbaker.

The results of our study and previous work suggest that the population of walleye in Lake Diefenbaker is in a hybrid zone, with a range of hybrid types, including those where introgression may render individuals indistinguishable from their parental species (Allendorf et al., 2001; Barton & Hewitt, 1985; Rhymer & Simberloff, 1996). This could lead to a hybrid swarm, which can alter the genetic composition of the parental species and reduce population and species differentiation, possibly leading to outbreeding depression (Allendorf et al., 2001; Mandeville et al., 2019; McFarlane & Pemberton, 2019; Rhymer & Simberloff, 1996; Scribner et al., 2001; Todesco et al., 2016; Weigel et al., 2003). In contrast, hybrid zones can also lead to novel genotypes and an increase in diversity within the species (Barton, 2001; Hamilton & Miller, 2015; Harrison & Larson, 2014; Seehausen, 2004). This uncertainty makes it important to understand the genomic impacts of hybridization to help mediate long‐term effects, particularly when hybridization is the result of anthropogenic habitat disturbances (Grabenstein & Taylor, 2018; Hasselman et al., 2014). This is especially true for fish in reservoirs, which are particularly susceptible to environmental change in combination with heavy anthropogenic impact (Hayes et al., 1999). Importantly, the impact of the hybridization documented in these lakes, if any, is unknown. Nevertheless, the presence of a hybrid swarm in Lake Diefenbaker indicates that hybridization is a pervasive and well‐established biological phenomenon in that waterbody. Reservoirs such as Lake Diefenbaker often support important recreational fisheries and/or provide broodstock for stocking programs; a decline in population productivity due to outbreeding depression may not only deteriorate the population, but also have larger socio‐economic impacts.

This study shows that it is crucial to include genetic assessments in fisheries management activities that require correctly identifying walleye, sauger, and their hybrids. Such activities may include broodstock collection, hybrid zone identification, and general population surveys. Overall, 7.0% of fish were misidentified morphologically based on broad species and hybrid categories, with levels of misidentification varying by reservoir. Most concerningly, several fish presumed to be pure walleye were identified genetically as F_1_ hybrids. F_1_ individuals should be phenotypically intermediate to parental species; however, our results and previous work suggest this is not always the case (Billington et al., 1997; Flammang & Willis, 1993; Van Zee et al., 1996; Ward & Berry, 1995; White et al., 2005). Further, parental species were misidentified as hybrids in the field, illustrating that natural phenotypic variation and introgressed markers in pure parental species may also be misleading. Our analyses of field‐identified walleye revealed hidden sauger introgression in 8% of fish based on crosses beyond F_1_ in Lake Diefenbaker. This finding indicates introgression within the walleye genome, which is common with backcrosses, especially after multiple generations, as individuals with low levels of admixture may not have intermediate morphology (Allendorf et al., 2001; McFarlane & Pemberton, 2019). Due to the hidden introgression detected in this study, fisheries managers should take caution, or entirely avoid sourcing broodstock from waterbodies where spontaneous hybridization is occurring. This is especially important when the purpose of the broodstock collection is for supplementing natural reproduction and/or stocking in locations with connectivity to waterbodies with self‐sustaining populations. However, the negative consequences of potential hybrid stocking in hydrologically isolated waterbodies, or waterbodies that lack suitable conditions for natural production (i.e., put‐take fisheries) are inherently lower. In Saskatchewan, this means that Lake Diefenbaker, Tobin Lake, and possibly other locations in the Saskatchewan River may not be suitable broodstock sources for all target waterbodies. Ultimately, it is vital to routinely monitor the genetic integrity of samples where spawn is collected in order to mitigate the risk of genetic introgression into broodstock and other systems. In addition, monitoring the genetic integrity of populations which were originally sourced from broodstock with potential introgression will aid in understanding the dispersal of hybrids through stocking activities and potential consequences.

## CONFLICTS OF INTEREST

The authors declare no conflict of interest.

## Supporting information

Supplementary Material

Supplementary Material

## Data Availability

All relevant data are available from Dryad at https://doi.org/10.5061/dryad.8cz8w9gnx (Graham et al., 2020).
