# Supplementary material for: Spontaneous hybridization and introgression between walleye (Sander vitreus) and sauger (Sander canadensis) in two large reservoirs: Insights from genotyping by sequencing
Source: Evol Appl. 2020 Dec 14;14(4):965–82. doi: 10.1111/eva.13174 (PMC8061268; doi:10.1111/eva.13174)
Supplement: Supplementary file 2 — Supplementary Material [file EVA-14-965-s002.docx]

**SUPPLEMENTARY MATERIAL**

**Table S1.** Pairwise fixation indices (F_ST_) output from GENODIVE with the dataset generated with the loci genotyped in 90% of the individuals (r90). F_ST_ values above the diagonal correspond to the FDR-corrected p-value below. The bolded numbers represent significant F_ST_ values (P<0.05). Site abbreviations can be found in Table 1.

|  | SGR-LD-R | SGR-LD-SL | SGR-TL | WALL-LD-CB | WALL-LD-R | WALL-LD-SL | WALL-TL |
| --- | --- | --- | --- | --- | --- | --- | --- |
| SGR-LD-R | -- | 0.006 | **0.021** | **0.858** | **0.772** | **0.813** | **0.818** |
| SGR-LD-SL | 0.214 | -- | **0.011** | **0.876** | **0.787** | **0.827** | **0.828** |
| SGR-TL | 0.001 | <0.001 | -- | **0.879** | **0.795** | **0.834** | **0.829** |
| WALL-LD-CB | 0.001 | <0.001 | <0.001 | -- | **0.028** | **0.030** | **0.061** |
| WALL-LD-R | <0.001 | <0.001 | <0.001 | 0.014 | -- | 0 | **0.042** |
| WALL-LD-SL | <0.001 | <0.001 | <0.001 | <0.001 | 0.331 | -- | **0.038** |
| WALL-TL | <0.001 | <0.001 | <0.001 | <0.001 | <0.001 | <0.001 | -- |

**Table S2.** Pairwise fixation indices (F_ST_) output from GENODIVE with the dataset generated with the loci genotyped in 100% of the individuals (r100). F_ST_ values above the diagonal correspond to the FDR-corrected p-value below. The bolded numbers represent significant F_ST_ values (P<0.05). Site abbreviations can be found in Table 1.

|  | SGR-  LD-R | SGR-  LD-SL | SGR-TL | WALL-LD-CB | WALL-LD-R | WALL-LD-SL | WALL-TL |
| --- | --- | --- | --- | --- | --- | --- | --- |
| SGR-  LD-R | -- | 0.001 | 0.023 | **0.878** | **0.802** | **0.846** | **0.861** |
| SGR-  LD-SL | 0.474 | -- | **0.017** | **0.893** | **0.815** | **0.858** | **0.868** |
| SGR-TL | 0.018 | 0.027 | -- | **0.886** | **0.817** | **0.857** | **0.866** |
| WALL-LD-CB | <0.001 | <0.001 | <0.001 | -- | **0.019** | **0.014** | **0.088** |
| WALL-LD-R | <0.001 | <0.001 | <0.001 | 0.043 | -- | 0.001 | **0.057** |
| WALL-LD-SL | <0.001 | <0.001 | <0.001 | 0.026 | 0.276 | -- | **0.056** |
| WALL-TL | <0.001 | <0.001 | <0.001 | <0.001 | <0.001 | <0.001 | -- |

**
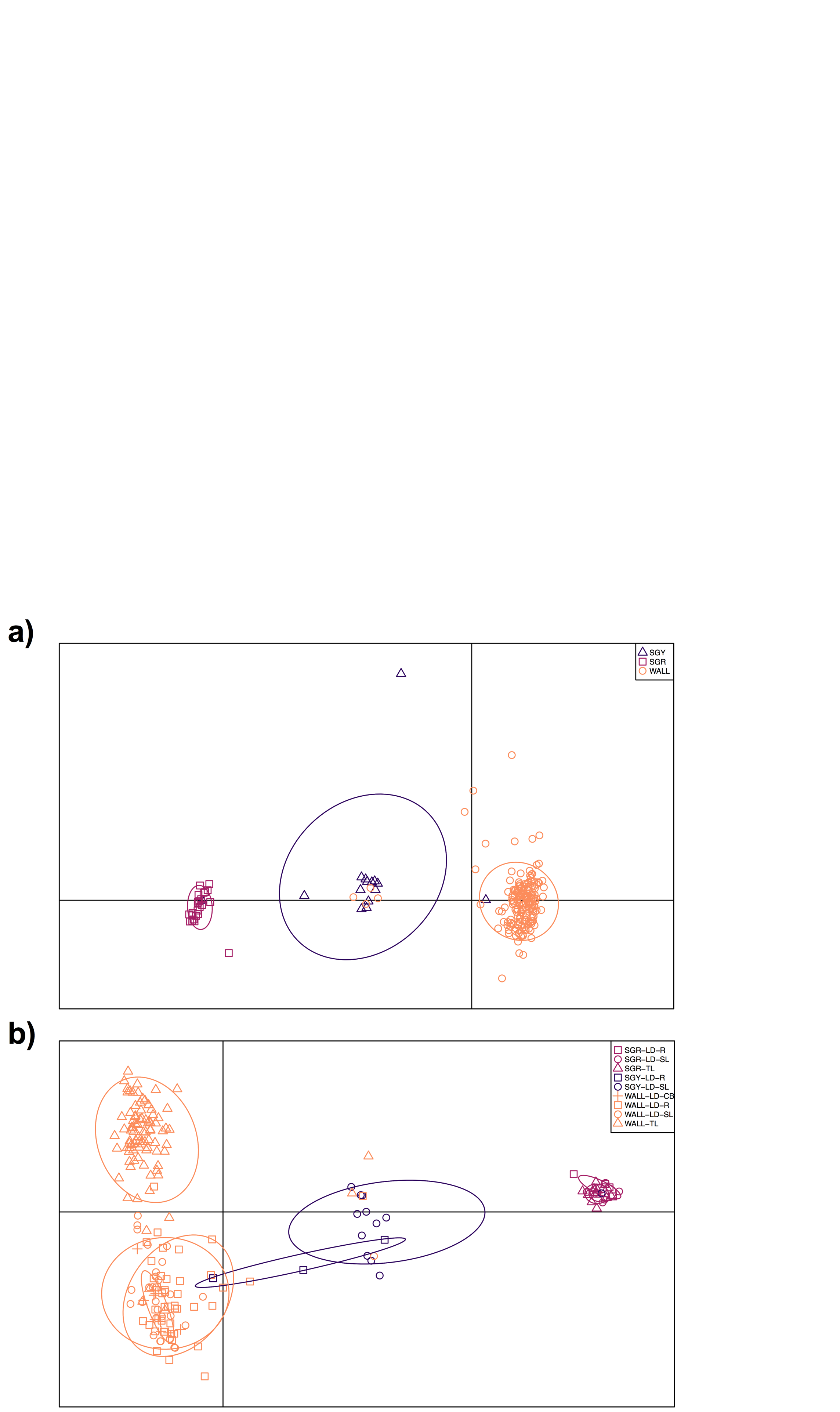
**

**Figure S1.** Discriminant analysis of principal components (DAPC) of the different species (a) and sample sites (b) in the r100 dataset. The DAPC analysis was run with 9 and 13 principal components in the species and sites analyses, respectively. Distinct ellipses indicate population differentiation. Site abbreviations can be found in Table 1.

**
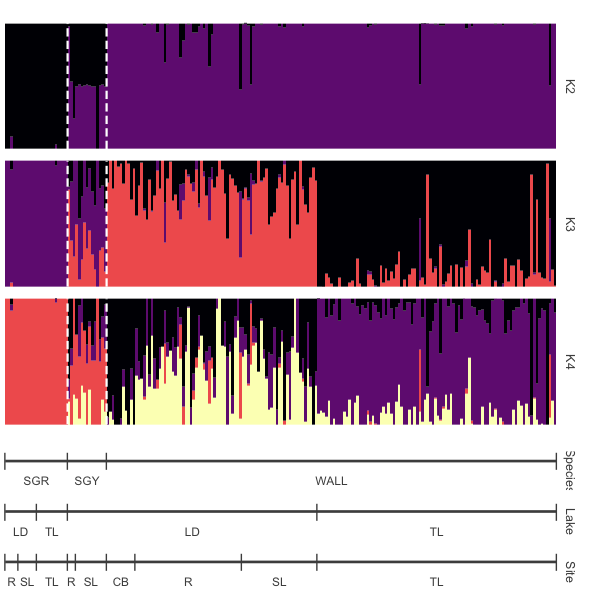
**

**Figure S2.** ADMIXTURE analysis across species from all sample sites in the r100 dataset. Each line represents an individual from the corresponding sample site. Site abbreviations can be found in Table 1.
